# Rare Case of Multifocal Spinal Hemangioblastomas With Holocord Syringo-Hydromyelia in an Adolescent Male

**DOI:** 10.7759/cureus.102651

**Published:** 2026-01-30

**Authors:** Rishi Meswani, Prashant Onkar, Suresh Phatak, Kajal Mitra, Mayank Rangari

**Affiliations:** 1 Radiodiagnosis, NKP Salve Institute of Medical Sciences & Research Centre, Nagpur, IND; 2 Radiodiagnosis, Datta Meghe Institute of Higher Education and Research, Sawangi, IND; 3 Radiodiagnosis and Imaging, NKP Salve Institute of Medical Sciences & Research Centre, Nagpur, IND; 4 Radiology, Manipal Hospitals, Bangalore, IND

**Keywords:** adolescent, avid homogeneous enhancement, flow voids, spinal hemangioblastoma, syringo-hydromyelia, von hippel-lindau syndrome

## Abstract

Hemangioblastomas are benign, highly vascular tumors of the central nervous system. Although they most commonly arise in the cerebellum, they may also involve the brainstem and spinal cord. The presence of multiple spinal hemangioblastomas is frequently associated with von Hippel-Lindau (VHL) syndrome; however, sporadic cases without an underlying genetic syndrome can occur. Characteristic imaging features include avid post-contrast enhancement, associated syrinx formation, and intratumoral or peritumoral flow voids. Spinal hemangioblastomas account for a small proportion of intramedullary spinal tumors and are rarely reported in adolescents. We present a rare case of multifocal spinal hemangioblastomas with extensive holocord syringo-hydromyelia in an adolescent male patient without any evidence of VHL syndrome.

## Introduction

Spinal hemangioblastomas are uncommon but highly vascular tumors, typically found within the intramedullary compartment of the spinal cord. They most often present between the third and fifth decades of life and are most commonly associated with von Hippel-Lindau (VHL) syndrome, which presents with multiple benign and malignant tumors involving multiple systems. However, sporadic cases can occur [1]. They are benign tumors which do not undergo malignant degeneration and are classified as WHO Grade I tumors [2]. The most common site is the cervical or thoracic spinal cord [3,4]. Hemangioblastomas show significant radiological overlap with other intramedullary tumors like ependymomas and astrocytomas. Therefore, MRI is necessary for characterization of these lesions. Radiologically, these tumors typically present as well-defined intramedullary lesions that can appear isointense to the spinal cord on T1-weighted images. But on post-contrast study, they show avid homogeneous enhancement and are often associated with extensive syrinx formation, which can result in significant neurological impairment. This characteristic enhancement is a hallmark feature of spinal hemangioblastoma due to its highly vascular parenchyma which consists of thin-walled, tightly packed blood vessels interspersed with stromal cells [5].

## Case presentation

A 17-year-old boy presented with a 10-day history of progressive left-sided weakness. He reported a brief episode of fever approximately 15 days prior to symptom onset, which was considered incidental, as there were no clinical or imaging findings suggestive of an inflammatory or infective etiology. Neurological examination revealed left-sided motor weakness with exaggerated deep tendon reflexes. There was no personal or family history suggestive of von Hippel-Lindau (VHL) syndrome.

**Figure 1:**
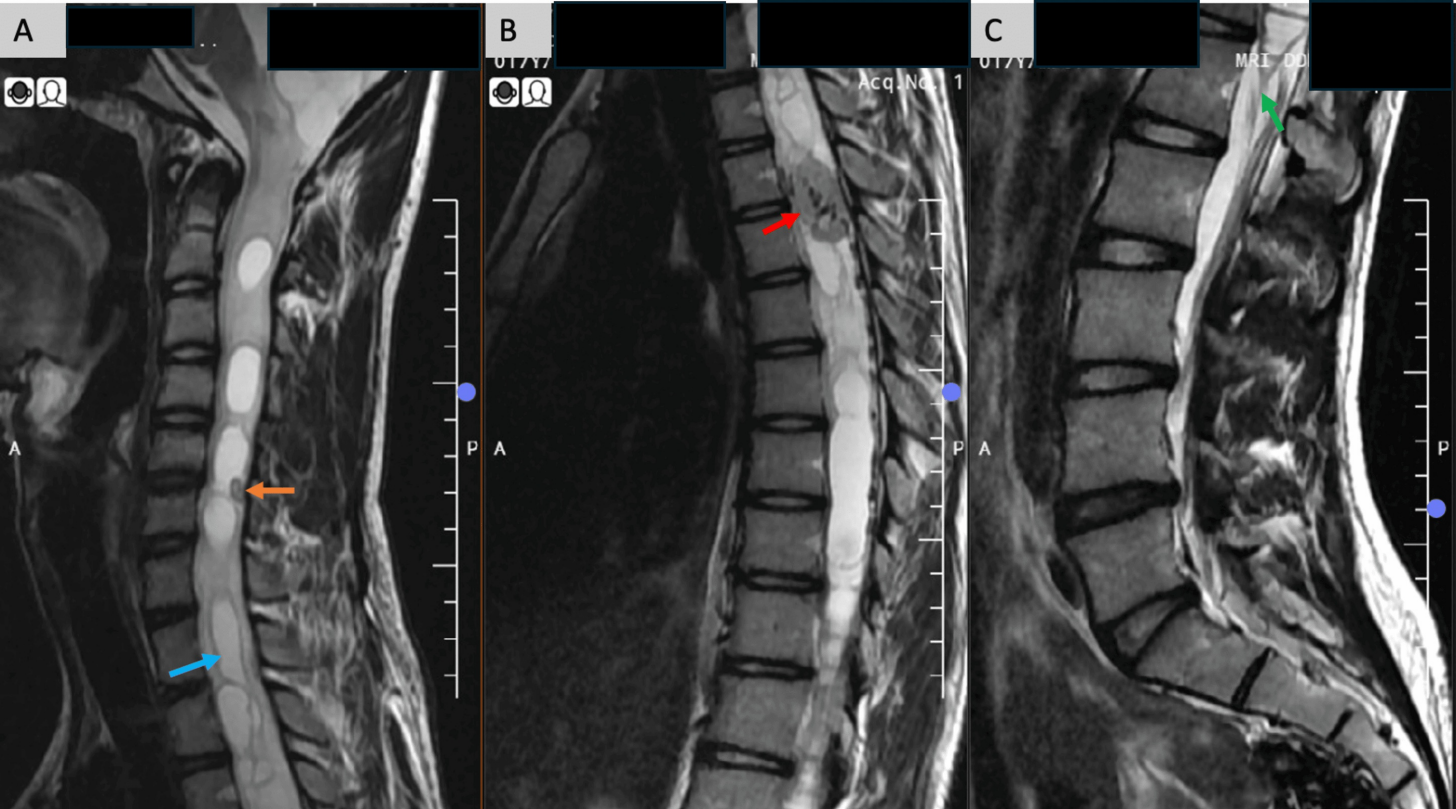
MRI sagittal T2-weighted image (A) Extensive syrinx formation (blue arrow) was seen extending superiorly up to the cervico-medullary junction, and a tiny T2-hypointense focus at C5-C6 level (orange arrow); (B) T2-hypointense intramedullary lesion with internal flow voids noted at the D4-D5 level (red arrow); (C) Inferior extension of the syrinx noted extending up to the level of conus (green arrow).

MRI of the spine demonstrated extensive holocord syringo-hydromyelia, extending from the cervical spinal cord inferiorly to the level of the conus (Figure 1). 

**Figure 2:**
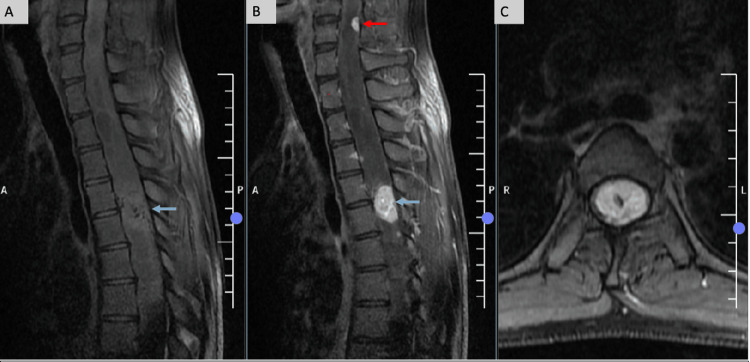
Pre- and post-contrast sagittal and axial T1-weighted image (A) Sagittal T1-weighted image showing an isointense intramedullary lesion at the D4-D5 level (blue arrow); (B) Avid, homogeneous post-contrast enhancement of the lesion (blue arrow) and an additional enhancing focus at the C5-C6 (red arrow); (C) Axial post-contrast T1-fat-suppressed image confirming the intramedullary location.

A tiny T2-hypointense nodular focus was also identified at the C5-C6 intervertebral level. A well-defined intradural intramedullary lesion was identified at the D4-D5 intervertebral level, measuring approximately 2.4 × 1.4 cm (craniocaudal × anteroposterior dimensions measured on sagittal images). On T2-weighted imaging, the lesion appeared hypointense with internal flow voids. On sagittal T1-weighted images, it demonstrated an isointense signal. Post-contrast sequences revealed avid, homogeneous enhancement of the D4-D5 lesion, along with an additional small enhancing nodular focus at the C5-C6 level. Axial post-contrast fat-suppressed images confirmed the intramedullary location of the thoracic lesion (Figure 2).

Based on the imaging findings of avidly enhancing intramedullary lesions with internal flow voids and extensive syringo-hydromyelia, spinal hemangioblastoma was considered the most likely diagnosis. Differential diagnoses included spinal astrocytoma and ependymoma, given the patient’s age and intramedullary location; however, the presence of intense homogeneous enhancement, prominent flow voids, and disproportionate syrinx formation favored hemangioblastoma.

The patient was evaluated for VHL syndrome using abdominal and pelvic ultrasonography and ophthalmologic examination, both of which were unremarkable.

**Figure 3:**
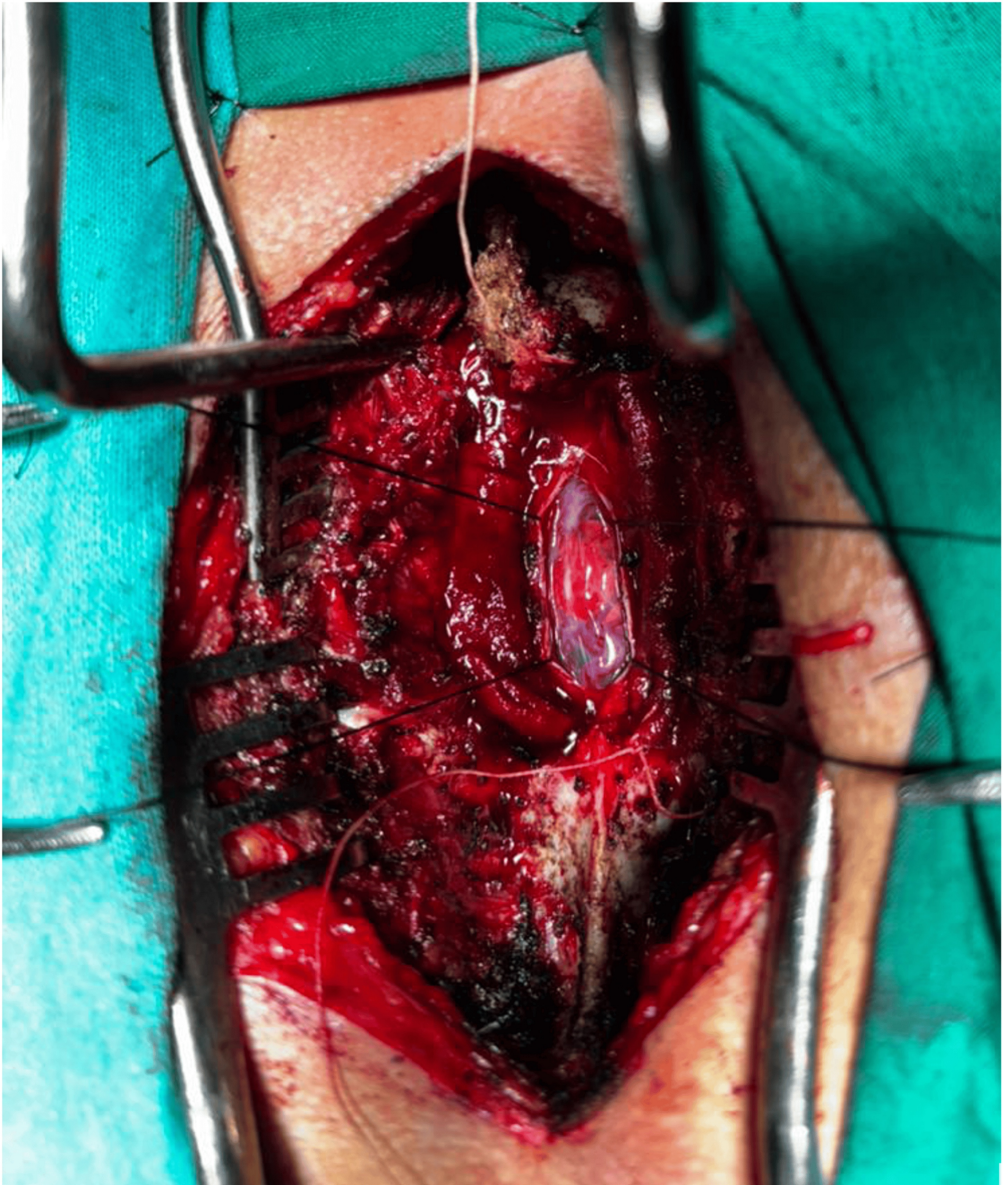
Intra-operative image Intraoperative photograph demonstrating the well-circumscribed, highly vascular reddish intradural tumor, consistent with hemangioblastoma.

He underwent a surgical resection of the D4-D5 lesion, which revealed a well-circumscribed, reddish, highly vascular intradural intramedullary tumor (Figure 3).

Histopathological examination demonstrated polygonal tumor cells with foamy cytoplasm, a delicate capillary network, areas of hemorrhage, and dilated vessels, confirming the diagnosis of hemangioblastoma. The smaller C5-C6 enhancing nodule was managed conservatively with close radiological surveillance. At the one-month follow-up, the patient demonstrated significant clinical improvement with marked reduction in left-sided weakness. 

## Discussion

Spinal hemangioblastomas are uncommon vascular tumors of the spinal cord that are considered benign, with no potential for malignant transformation. They fall under the WHO grade I classification [2]. These tumors may occur sporadically or in association with VHL syndrome, particularly when multiple small lesions are present.

The most common age group for these tumors is 30 to 65 years, and they show a slight male predominance [6]. Both VHL-associated and sporadic hemangioblastomas are extremely rare in children and adolescents, with an incidence  of <1:1,000,000 [7]. Large, spinal hemangioblastomas tend to present with symptoms that are typical of other spinal cord tumors, such as sensory or motor deficits and localized pain. On MRI, they typically appear hypo- to isointense on T1-weighted images and iso- to hyperintense on T2-weighted sequences, relative to the spinal cord, with the presence of prominent flow voids, which makes them appear heterogeneous. Post-contrast T1-weighted imaging with gadolinium usually reveals uniform and strong enhancement. Smaller lesions may mimic the appearance of normal spinal cord tissue, making them harder to distinguish. These tumors usually have well-defined margins and are most often found on the posterior side of the spinal cord, located superficially due to their subpial position. Despite being small in size, they are frequently associated with disproportionately large syrinx formation and prominent vascular flow voids. In fact, around 50-70% of cases show syrinx development, which is thought to result from fluid secretion by the tumor and leakage from its vascular channels [6]. Although the intramedullary location is most common, spinal hemangioblastomas may also appear in the intradural extramedullary or even extradural compartments [8,9].

In the present case, two intramedullary lesions were seen at the C5-6 and D4-5 intervertebral level, appearing iso- to hypointense on T1-weighted, T2-weighted, and short Tau inversion recovery (STIR) images with internal flow voids. Extensive syringo-hydromyelia was noted extending from the ponto-medullary junction down to the conus at L1. The cystic component in the medulla showed an exophytic extension into the inferior cerebellar cistern. On the post-contrast sequences, the two intramedullary lesions showed avid homogeneous enhancement.

## Conclusions

Spinal hemangioblastomas are rare, benign, and highly vascular intramedullary tumors, particularly uncommon in the adolescent age group. When present, they characteristically demonstrate avid homogeneous enhancement, intratumoral or peritumoral flow voids, and are frequently associated with disproportionate syrinx formation. This case highlights a rare presentation of multifocal spinal hemangioblastomas with extensive holocord syringo-hydromyelia in an adolescent patient without clinical or systemic evidence of VHL syndrome.

Although multiple spinal hemangioblastomas are classically associated with VHL syndrome, this report underscores that sporadic multifocal disease can occur even in the absence of an underlying genetic disorder. Awareness of this possibility is important to avoid diagnostic delay or misclassification as other intramedullary tumors such as astrocytoma or ependymoma. Early and accurate radiological recognition plays a crucial role in guiding appropriate surgical management, surveillance strategies for additional lesions, and optimizing neurological outcomes in this rare yet clinically significant entity.
